# The Formation Mechanism of Defects, Spiral Wave in the Network of Neurons

**DOI:** 10.1371/journal.pone.0055403

**Published:** 2013-01-31

**Authors:** Xinyi Wu, Jun Ma

**Affiliations:** Department of Physics, Lanzhou University of Technology, Lanzhou, China; University of Pittsburgh, United States of America

## Abstract

A regular network of neurons is constructed by using the Morris-Lecar (ML) neuron with the ion channels being considered, and the potential mechnism of the formation of a spiral wave is investigated in detail. Several spiral waves are initiated by blocking the target wave with artificial defects and/or partial blocking (poisoning) in ion channels. Furthermore, possible conditions for spiral wave formation and the effect of partial channel blocking are discussed completely. Our results are summarized as follows. 1) The emergence of a target wave depends on the transmembrane currents with diversity, which mapped from the external forcing current and this kind of diversity is associated with spatial heterogeneity in the media. 2) Distinct spiral wave could be induced to occupy the network when the target wave is broken by partially blocking the ion channels of a fraction of neurons (local poisoned area), and these generated spiral waves are similar with the spiral waves induced by artificial defects. It is confirmed that partial channel blocking of some neurons in the network could play a similar role in breaking a target wave as do artificial defects; 3) Channel noise and additive Gaussian white noise are also considered, and it is confirmed that spiral waves are also induced in the network in the presence of noise. According to the results mentioned above, we conclude that appropriate poisoning in ion channels of neurons in the network acts as ‘defects’ on the evolution of the spatiotemporal pattern, and accounts for the emergence of a spiral wave in the network of neurons. These results could be helpful to understand the potential cause of the formation and development of spiral waves in the cortex of a neuronal system.

## Introduction

Beautiful spatiotemporal patterns can be observed in reaction-diffusion systems [1]–[13] and networks of coupled oscillators and/or neurons [14]–[25]. The appearance of these various patterns is often associated with the self-organization and competition among cells in the nonlinear system. Spiral waves emerge in a spatiotemporal system when not in a thermodynamical equilibrium state. The dynamics of a spiral wave in a reaction-diffusion system has been investigated extensively, and it is confirmed that the formation of a spiral wave in cardiac tissue could be associated with specific types of arrhythmias (reentrant ventricular tachycardia and ventricular fibrillation) [26], [27]. Some reliable theoretical models [28]–[31] have been proposed to measure the dynamics of spiral waves, and some feasible schemes [32]–[36] have been presented to suppress the spiral wave turbulence, and to prevent the breakup [37] of spiral waves. Spiral waves and target waves [38]–[41] are ordered waves, and these waves are often observed in the excitable media. Generally, a target wave could be induced to occupy the media or network when appropriate periodical forcing [33]–[35] is imposed on the media in a local area, and it also could be induced by local heterogeneity [39] or local feedback control. Zhang et al. [42] suggested that local periodic forcing could suppress spiral waves and spatiotemporal chaos by generating target wave in the media. Liu et al. [43] reported that local diversity in parameters could induce a target wave, and local self-coupling feedback [44], [45] can suppress the spiral wave when a target wave is developed. Qian et al. [46] discussed the formation of self-sustained target wave in excitable media with only one long-range connection. Gao et al. [47] confirmed that block variable (phase compress) can generate a target wave in an oscillatory media. Aranson et al. [48] reported the motion of a spiral wave in an excitable media due to interaction with various kinds of boundaries. Zykov [49] revealed the selection principle, which determines the shape and the rotation frequency of spiral waves in an unbounded medium with a given excitability.

A spiral wave could also be developed in a sub-excitable medium [50] when noise is imposed on the medium. Electric activities of neurons show distinct regularity due to coherence resonance under optimized noise. For example, Tang et al. [24] investigated the dynamics of a spiral wave under multiple spatial coherence resonances by introducing colored noise into the network. Gu et al. [25] investigated the selection of a spiral wave in a network of Morris-Lecar (ML) neurons with type I excitability when multiple spatial coherence resonances emerge in the network. These results help to explain the emergence of spiral waves in the mammalian middle cortex and visual cortex [51]–[53], which these experimental results showed that these spiral waves could regulate the wave propagation among neurons as a pacemaker. In fact, spiral waves can be observed in highly unnatural experimental conditions (disinhibited cortical slices) or some pathological states. However, cortical electrical activity does not show spirals in physiological conditions.

Furthermore, the transition of a spiral wave in a network of Hodgkin-Huxley neurons induced by diffusive poisoning [54] in ion channels, and due to changeable probability [55] of long-range connection is investigated, respectively.

In numerical studies, specific initial values are often used to generate a spiral seed so that a stable rotating spiral wave could be developed for further study. In fact, the spiral wave is dependent on the tip, which is associated with a topology defect. A spiral wave could also be induced by breaking a travelling wave. For example, Cai et al. [56] reported that a spiral wave could be formed in a sub-excitable medium by breaking the plane wave caused by the presence of a suitable electric field. Spiral waves are often illustrated by calculating the spatial distribution of measurable variables such as the membrane potential of neurons in the network. It is convenient to measure the statistical properties of the collective electric activities of neurons or coupled oscillators. For example, Gonze et al. [57] defined a statistical factor in one-dimensional space to measure the synchronization degree in coupled circadian oscillators. Refs. [54], [55] redefined this statistical variable in two-dimensional space to measure the transition of a spiral wave in the network induced by bifurcation parameter.

In contrast to the results in Refs. [24], [25], this paper will discuss 1) the formation of a target wave in the network of improved Morris-Lecar neurons [58]–[60] induced by forcing current with diversity; 2) the formation of a spiral wave by blocking a target wave in two different ways, generating artificial defects and simulating partial ion channels blocking; 3) Channel noise and additive noise are also considered. The potential mechanism for the origin of defects in a realistic neuronal system will be discussed. The simulations confirm that the emergence of a spiral wave in the network induced by breaking a target wave with defects (artificial defects or a poisoned area) keeps certain robustness to channel noise or additive Gaussian white noise.

### Models and the Scheme

The dynamical equations for the two-dimensional network of Morris-Lecar (ML) neurons with nearest-neighbor connection [24], [25] are described as follows
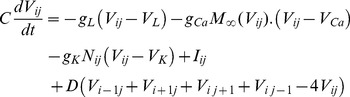
(1a)

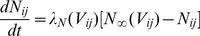
(1b)

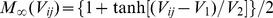
(2a)

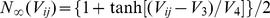
(2b)

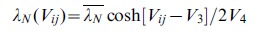
(2c)


The activator *V_ij_* represents the membrane potential of neuron in node (*i*, *j*) with the physical unit *mV*, and *N* denotes the gate variable for potassium channel. The parameters *g*_Ca_, *g*_K_, *g*_L_ are the conductance associated with the three transmembrane currents, the corresponding maximal conductance are marked with 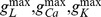; *V*_Ca_, *V*_K_, and *V*_L_ are the corresponding reversal potentials. Parameter *C* (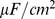) defines the capacitance per unit area for the membrane of the neuron, and *I_ij_* measures the external forcing current on the neuron in node (*i*, *j*). Noise can change the excitability and the fluctuation of the membrane potential of neurons, and thus the electric activity of the neurons is adjusted. Generally, channel noise and/or additive Gaussian white noise is introduced into the neuron models. The dynamical equation for the gate variable under channel noise is often described as follows
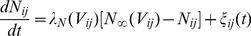
(3)


While the membrane potentials of neurons are still measured by Eq. (1a), the statistical properties of Gaussian white noise *ξ* (*t*) [61], [62] on a single neuron is given by
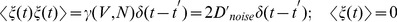
(4a)

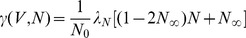
(4b)where 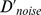 is the intensity of channel noise, *N*_0_ is the number of working and open channels, the random variable *ξ* (*t*) is defined as 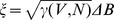. 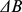 is regarded as an increment for the Wiener process (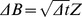, Z∼*N* (0,1) ). According to Eq. (4a, 4b), the intensity of noise on different nodes could be in difference. The statistical properties of noise on any node (*i, j*) in the network could be approached by introducing subscript (*i, j*) into the variables *N, V* in Eq. (4a, 4b) as *N_ij_, V_ij_*. As a result, a stochastic ML model is redefined as follows



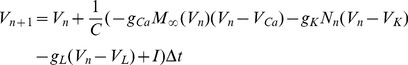
(5a)

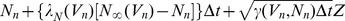
(5b)


The redefined dynamical equations for the network of ML neurons could also be approached by marking subscripts (*i, j*) on the variables *V*_n_, *V*_n+1_, *N*_n_, *N*_n+1_, *I*. On the other hand, membrane potentials of neurons could also be perturbed by other unknown factors, and additive Gaussian white noise is often used to represent the effect on the fluctuations of membrane potential. The dynamical equation for a network of ML neurons in the presence of additive Gaussian white noise [25] is described as follows
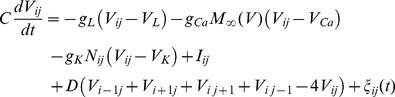
(6)


The gate variable *N_ij_* still is defined as shown in Eq. (1b). The statistical properties [25] of the additive Gaussian white noise *ξ_ij_* (*t*) on node (*i, j*) is defined as follows.
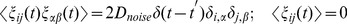
(7)


where *D*_noise_ is the intensity of additive Gaussian white noise on a single neuron, *δ* (*) represents the *Dirac*-*δ* function, *δ_i,_*_α_ = 1 for *α = i*; *δ_i,_*_α_ = 0 for *α*≠*i; δ_j_*_,β_ = 1 for *β = j*; *δ_j_*_,β_ = 0 for *β*≠*j*. In a realistic neuronal system, a large number of neurons are included in the functional domain or a local area. It is better to detect the main properties of electric activities of neurons; a statistical variable is defined to detect the collective behavior of neuronal membrane potential. In Ref. [57], a statistical factor of synchronization is defined in one-dimensional space to detect the distinct shift of oscillators according to the theory of mean field. The statistical factor is defined in the two-dimensional space as follows
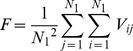
(8a)

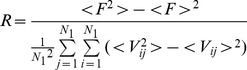
(8b)where the variable *F* is the averaged instantaneous membrane potentials of all neurons in the network, *N*_1_^2^ is the number of nodes, the factor of synchronization *R* is calculated over all neurons with certain transient period, a perfect synchronization state is reached for *R*∼1 while no synchronization is found at *R*∼0. Some of previous works confirmed that a smaller value for the factor of synchronization is associated with the emergence of a spiral wave in the network.

A spiral wave in the media could be initiated by setting appropriate initial values, or breaking the travelling wave. In Ref. [55], we confirmed that a spiral wave could also be induced to occupy the network when the target wave is blocked by the defects in a brief way. However, these defects are artificial defects and the formation mechanism for these defects should be investigated extensively. At first, we discuss the formation and development of a spiral wave by blocking the target wave in the network with artificial defects. Two critical steps are required as follows.

1. A stable target wave is developed in the network with external forcing currents with diversity imposing on some neurons in a local area of the network. External forcing current with intensity *I*_0_ is imposed on the neurons in a square array with *S* = m*m neurons being included, the other neurons in the network of neurons experience an imposed forcing current with intensity *I*_1_. A target wave could be induced to occupy the network under appropriate diversity of forcing current *I*_0_−*I*_1_, and target wave will propagate from the small square array to other nodes in the network.
2. Artificial defects are made. The variables of neurons in another local area (aside from the source site for target wave) are set to zero.

In the numerical studies as presented in the next section, extensive numerical studies will be analyzed to investigate the development of a spiral wave when the target wave is blocked by the artificial defects in the network.

The fluctuation of membrane potentials of neurons could be changed by ion channel blocking, for example, some drugs can poison ion channels and thus some channels are blocked. Therefore, it is interesting to investigate the effect of partial closure of ion channels of some neurons in local area of the network on the target wave, and it is instructive to find if a spiral wave can also be initiated when the target wave is blocked by the poisoned area in the network. These results are helpful to understand the origin of defects in the neuronal network because the formation of a spiral wave is often associated with the appearance of defects close to the tip. The conductance is often changed when the ion channels are blocked, then the modified conductance [63] is given by
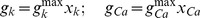
(9)where *x*_k_ (and *x*_Ca_) represents the ratio of working ion channels of potassium (and calcium) to the total ion channels of potassium (and calcium). A smaller value for *x*_k_ (and *x*_Ca_) indicates that only a small fraction of ion channels are working and open, while a bigger value for *x*_k_ (and *x*_Ca_ ) means that the ion channels are mostly open. In the following section, we mainly investigate the effect of ion channel blocking of potassium channels in neurons.

### Numerical Results and Discussion

In this section, the conductance and parameters in the network of neurons are selected as *g_l_* = 2.0,*g_ca_* = 4.0,*g_k_* = 8.0, *V*_L_ = −60.0,*V*_Ca_ = 120.0, *V*_K_ = −80.0, *V*_1_ = −1.2,*V*_2_ = 18.0, *V*_3_ = 12.0, *V*_4_ = 17.4, *C* = 5.0,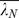  = 1/15. The Morris-Lecar model will be in type II excitability by selecting the parameters as above, which is much different from the parameter region in Ref. [25] in type I excitability. The time step is 0.001, network size is measured by 200×200 nodes and a no-flux boundary condition is used for the network. At first, we investigate the case of blocking target waves by using artificial defects. External forcing currents with diversity are imposed on the network, for simplicity, external forcing current *I*_0_ is imposed on the neurons in a square area (*S*) close to the center of the network, while another forcing current *I*_1_ is imposed on the neurons in the other nodes of the network. In this way, a gradient forcing current Δ*I* = *I*_0_−*I*_1_ is generated to change the membrane potentials of neurons in the network. As a result, a target wave could be developed with a certain transient period. The local area forced with current *I*_0_ consist of a number of neurons *S* = 9, 16, 25, 36, 49, and *I*_0_ = 48, 49, 50, 51, 52, 53, 54, 55 will be investigated, respectively. The neurons outside of the local square area (*S*) will be given the forcing current *I*_1_ = 40 if no special statement is presented.

**Figure 1:**
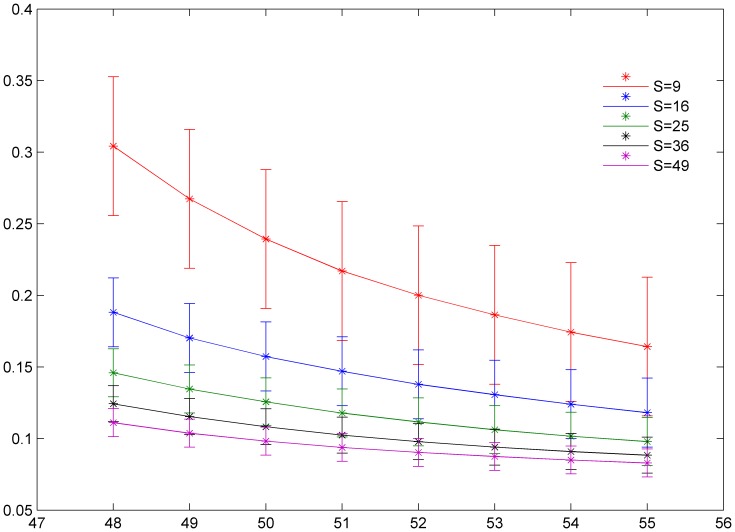
The distribution of factor of synchronization vs. forcing currents with diversity. The size of local area forced with current *I*_0_ is marked as *S* = 9 (90≤*i* ≤92, 90≤*j* ≤92), *S* = 16 (90≤*i* ≤93, 90≤*j* ≤93), *S* = 25 (90≤*i* ≤94, 90≤*j* ≤94), *S* = 36 (90≤*i* ≤95, 90≤*j* ≤95), *S* = 49 (90≤*i* ≤96, 90≤*j* ≤96), coupling intensity *D* = 4 and transient period is about 800 time units.

**Figure 2:**
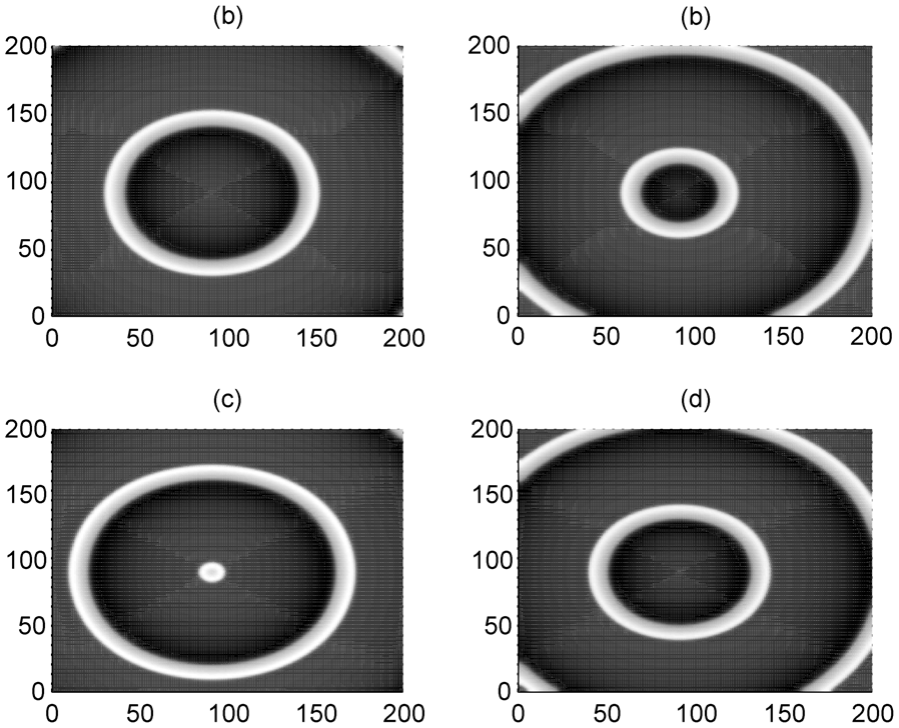
The formed target wave induced by forcing currents with diversity. The coupling intensity *D* = 4, S = 3×3 (90≤*i* ≤92, 90≤*j*≤92), *I*_1_ = 40. for *I*_0_ = 49 (panel a), *I*_0_ = 51 (panel b), *I*_0_ = 53 (panel c), *I*_0_ = 55 (panel d), transient period *t* = 800 time units and no-flux boundary condition is used.

**Figure 3:**
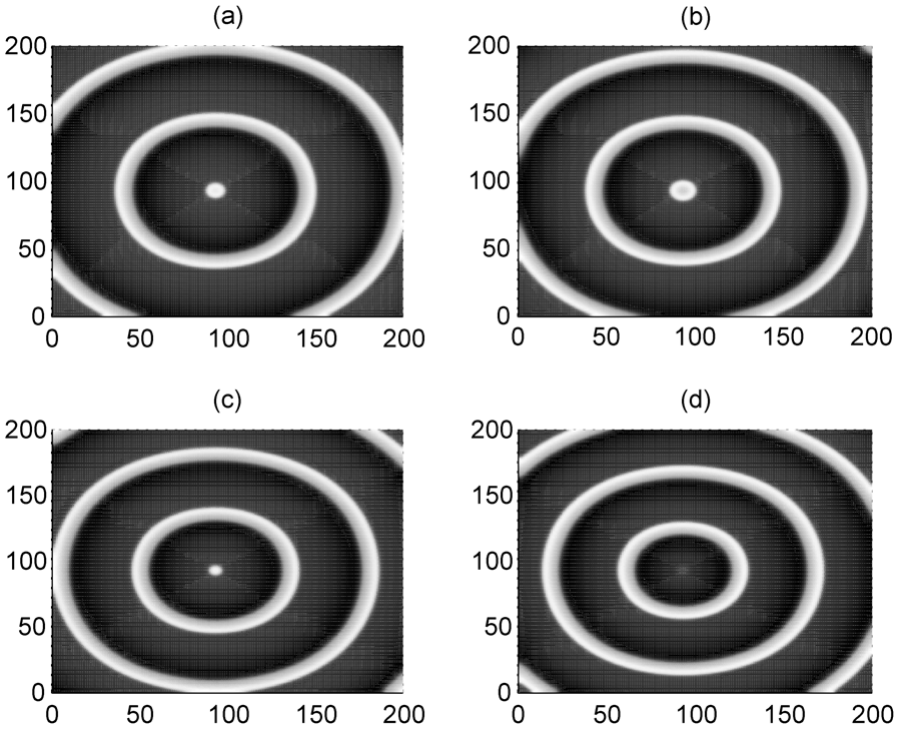
The formed target wave induced by forcing currents with diversity. The coupling intensity *D* = 4, S = 7×7 (90≤*i* ≤96, 90≤*j*≤96), *I*_1_ = 40, for *I*_0_ = 49 (panel a), *I*_0_ = 51 (panel b), *I*_0_ = 53 (panel c), *I*_0_ = 55 (panel d), transient period *t* = 800 time units and no-flux boundary condition is used.

The results in Fig. 1 show that the factor of synchronization decreases to a smaller value with increasing forcing current *I*_0_, and a smaller factor of synchronization is also detected when the size of the square area forced with current *I*_0_ is increased. An ordered state such as a target wave could be developed when the factor of synchronization is close to a smaller value. The potential cause is that a higher gradient Δ*I* = *I*_0_−*I*_1_ (*I*_1_ = 40) and larger size of square area excited by forcing current *I*_1_ make the adjacent neurons become excited and a travelling wave is propagated symmetrically to activate more neurons, so that a target-like wave is generated to occupy the network. To check the wave formation in a visualized way, some snapshots are presented in Fig. 2 and Fig. 3 to illustrate the distribution of membrane potentials of the neuron in the network at fixed time.

The results in Fig. 2 and Fig. 3 confirm that a target wave could be induced to occupy the network completely when the gradient current Δ*I* = *I*_0_−*I*_1_ (*I*_1_ = 40) and/or size of the square array forced by current *I*_0_ is not very small. It is also found that the sampled time series of membrane potentials show distinct periodicity when target wave is observed in the network. The developed target wave becomes dense when the number of neurons in the square array with network size S = m*m is increased. Nevertheless, target wave becomes sparse and even could fail to occupy more area of network when the network size S = m*m and/or gradient current Δ*I* = *I*_0_−*I*_1_ is decreased greatly.

**Figure 4:**
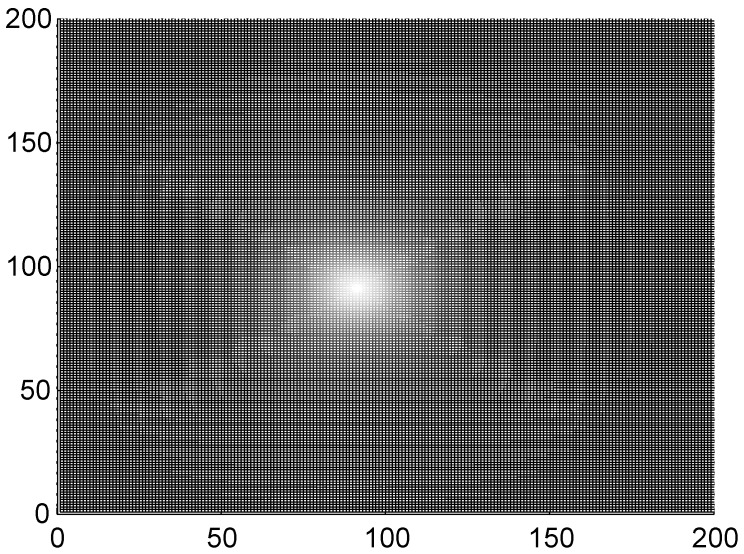
The formed target wave induced by forcing currents with diversity. The forcing current *I*_0_ = 41, coupling intensity *D* = 4, S = 3×3 (90≤*i* ≤92, 90≤*j*≤92), *I*_1_ = 40, transient period *t* = 800 time units and no-flux boundary condition is used.

**Figure 5:**
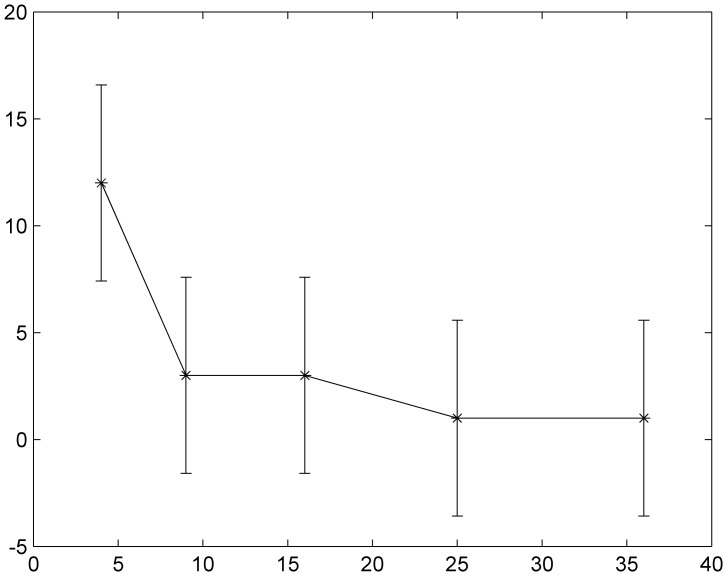
The distribution of forcing currents with diversity vs. forcing area for generating a target wave. The threshold for initiating a target wave in the network in the two-parameter space for square size forced by current *I*_0_ and gradient current Δ*I* = *I*_0_−*I*_1_.

The result in Fig. 4 confirmed that no distinct target wave could be found in the network while the gradient forcing Δ*I* = *I*_0_−*I*_1_ = 1 just induces target-like wave in a very small area, the reason could be that smaller forcing current *I*_0_ cannot activate the adjacent neurons, and thus no travelling wave is propagated to other neurons in the network. Furthermore, we calculated the critical threshold for developing a target wave in the network and the results are shown in Fig. 5.

The results in Fig. 5 show that higher gradient current is necessary when the square size forced by current *I*_0_ is small (fewer neurons are excited by current *I*_0_), otherwise, more neurons should be excited with current *I*_0_ synchronously.

**Figure 6:**
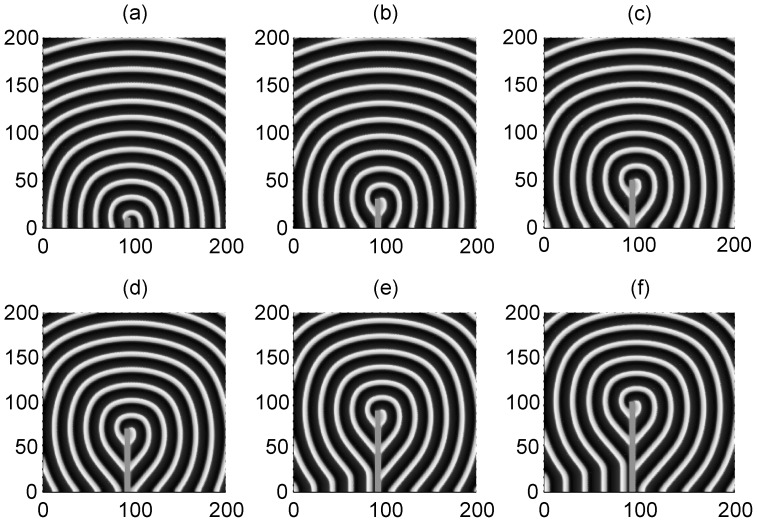
Formation of spiral waves induced by different artificial defects. The developed spiral wave by breaking the target wave via artificial defects with different sizes. The area for defects is within a rectangular area for 90≤*i* ≤95, 1≤*j* ≤10 (panel a), 90≤*i* ≤95, 1≤*j* ≤30 (panel b), 90≤*i* ≤95, 1≤*j* ≤50 (panel *c*) ,90≤*i* ≤95, 1≤*j* ≤70 (panel d), 90≤*i* ≤95, 1≤*j* ≤90 (panel *e*), 90≤*i* ≤95, 1≤*j* ≤100 (panel *f*). The variable for membrane potentials of neurons in this area are set as zero, the forcing currents with diversity are selected as *I*_0_ = 60 and *I*_1_ = 4, coupling intensity *D* = 2, transient period *t* = 800 time units and no-flux boundary condition is used.

The results in Fig. 6 show that a spiral wave could be induced and developed to occupy the network and the profile of the spiral waves are dependent on the size of the artificial defects. Extensive numerical studies confirm that a spiral wave will become sparse with increasing the coupling intensity. And the sampled time series of membrane potentials of neurons show distinct periodicity close to the intrinsic frequency of these stable rotating spiral waves. That is to say, these artificial defects could be helpful to induce spiral waves by breaking an ordered travelling wave such as a target wave.

**Figure 7:**
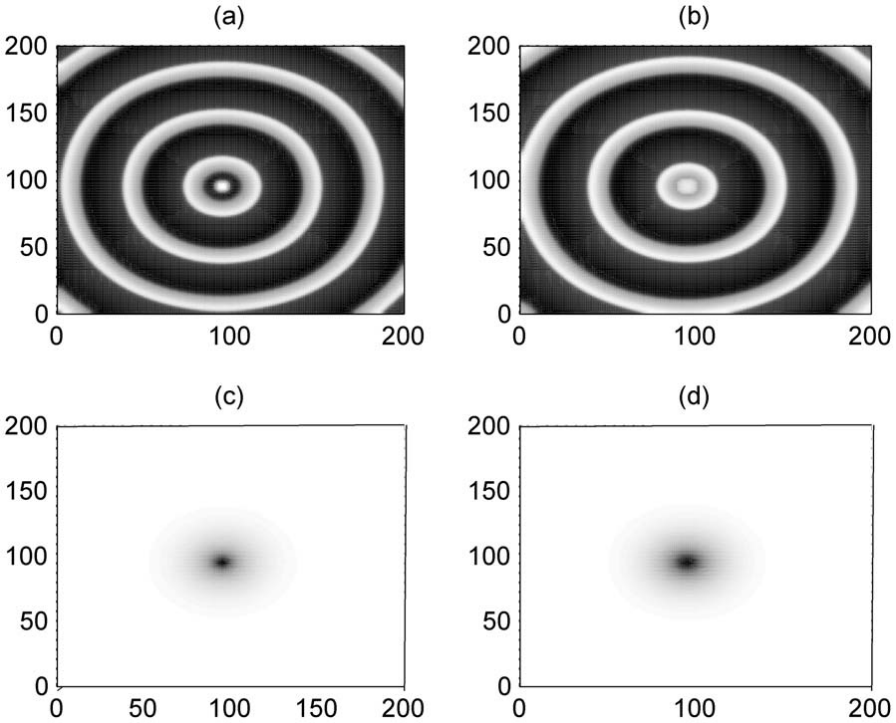
Formation of target wave induced by ion channels blocking in potassium. The developed state (*t* = 800 time units) when some potassium ion channels in a square array (90≤*i* ≤100, 90≤*j* ≤100) are blocked at *x*_Ca_ = 1, coupling intensity *D* = 4, for *x*_k_ = 0.1 (panel a), *x*_k_ = 0.2 (panel b),*x*_k_ = 0.3 (panel c), *x*_k_ = 0.4 (panel d).

However, it is more important to explore the occurrence of these suggested artificial defects. It has been confirmed that the excitability of neurons and electric activities of neurons could be changed due to the effect of ion channel poisoning or blocking when some drugs are absorbed in the neuronal or blood system. First, we discuss the case about poisoning and blocking of potassium ion channels in a small square array, and then the size of poisoned areas is increased as a rectangular array. In Fig. 7, we give an example to illustrate the formation of a target wave when the ion channels of few neurons in a local square array are blocked.

The results in Fig. 7 confirm that target wave could be induced when potassium channels in a few neurons are blocked with smaller parameter ratio *x*_k_ (depth poisoning). Otherwise, the target wave is compressed in a small area and could not occupy the network completely. The potential mechanism for the formation of a target wave in this case could be that neurons in the local poisoned area excited the adjacent neurons synchronously and generate pacemaker-like signals, and thus a target wave could be induced in the network. On the other hand, slight poisoning of the ion channels seldom generates distinct gradient forcing, thus no target wave is excited.

**Figure 8:**
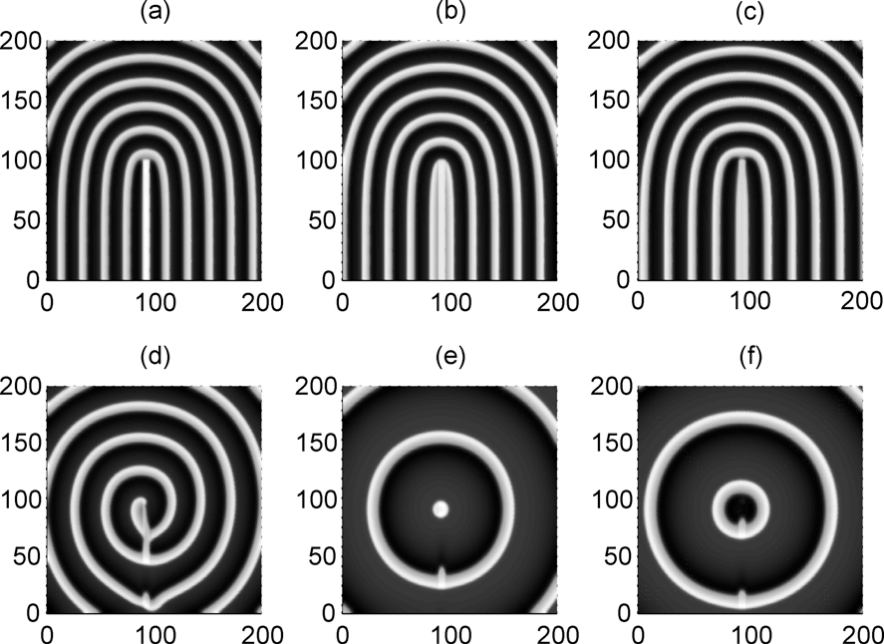
The interaction between the target wave due to forcing currents with diversity and the local poisoned area. The poisoned area for potassium ion channels is marked with 90≤*i* ≤95, 1≤*j* ≤100, for *x*_k_ = 0.1 (panel a), *x*_k_ = 0.2 (panel b), *x*_k_ = 0.3 (panel c), *x*_k_ = 0.4 (panel d), *x*_k_ = 0.5 (panel e), *x*_k_ = 0.6 (panel *f*) External forcing currents with diversity are imposed as *I*_0_ = 55 on the nodes within 90≤*i* ≤93, 1≤*j* ≤93, *I*_2_ = 40, coupling intensity *D* = 4,transient period *t* = 800 time units and no-flux boundary condition is used.

Furthermore, we check the case when the target wave induced by forcing currents with diversity is blocked or broken by the local poisoned area in the network, and the results are plotted in Fig. 8.

**Figure 9:**
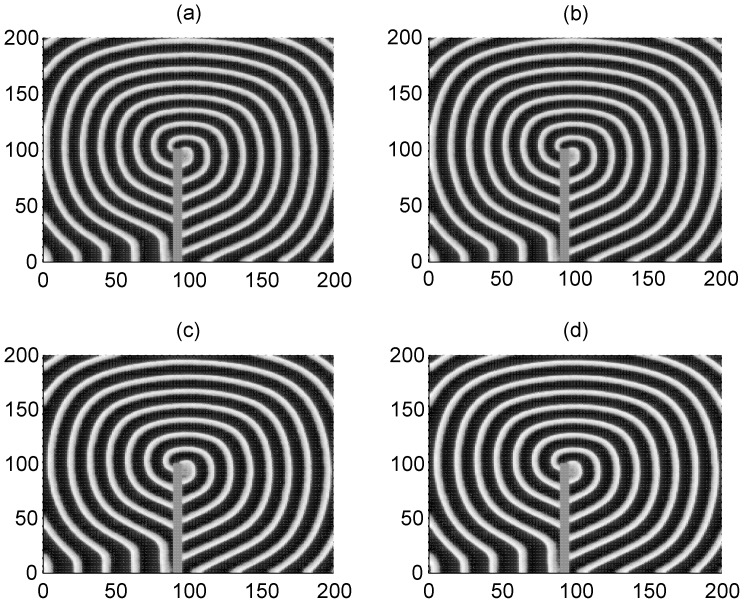
The development of spiral wave under different channel noise when the target wave is broken by artificial defects. Artificial defects area is within 90≤*i* ≤95, 1≤*j* ≤100 (*V_ij_* = 0). The area forced by external currents with diversity is close to the center of the network (90≤*i* ≤100, 90≤*j* ≤100). For *I*_0_ = 90,*N*_0_ = 200 (panel a) ; *I*_0_ = 90,*N*_0_ = 250 (panel b) ; *I*_0_ = 110,*N*_0_ = 200 (panel c) ; *I*_0_ = 110,*N*_0_ = 250 (panel d) ; at *I*_2_ = 40, coupling intensity *D* = 2, transient period *t* = 800 and no-flux boundary condition is used.

The results in Fig. 8 show that a spiral wave could be induced (as shown in Fig. 9d) when the target wave (induced by forcing current with diversity) is broken and blocked by the local poisoned area with appropriate parameter ratio *x*_k_. The developed target wave survives and can suppress the negative effect due to ion channel block in the local area under higher ratio *x*_k_ (slight poisoning in channels).

Clearly, the poisoned area plays similar role in breaking the target wave to initiate a spiral wave as artificial defects do. It is argued that the realistic defects in the network could result from the effect of channel block because the effect of ion channel blocking in neurons in local area is very similar to the effect of artificial defects on blocking the target wave to induce a spiral wave. However, it is important to check this conclusion when channel noise is considered.

As mentioned above, it is interesting to investigate the effect of channel noise on the formation of a spiral wave when the target wave is blocked by the poisoned area in the network. The channel noise is calculated using Eq. (4). First, we investigate the case when spiral wave is formed by blocking the target wave with artificial defects in the presence of channel noise, and the results are plotted in Fig. 9.

**Figure 10:**
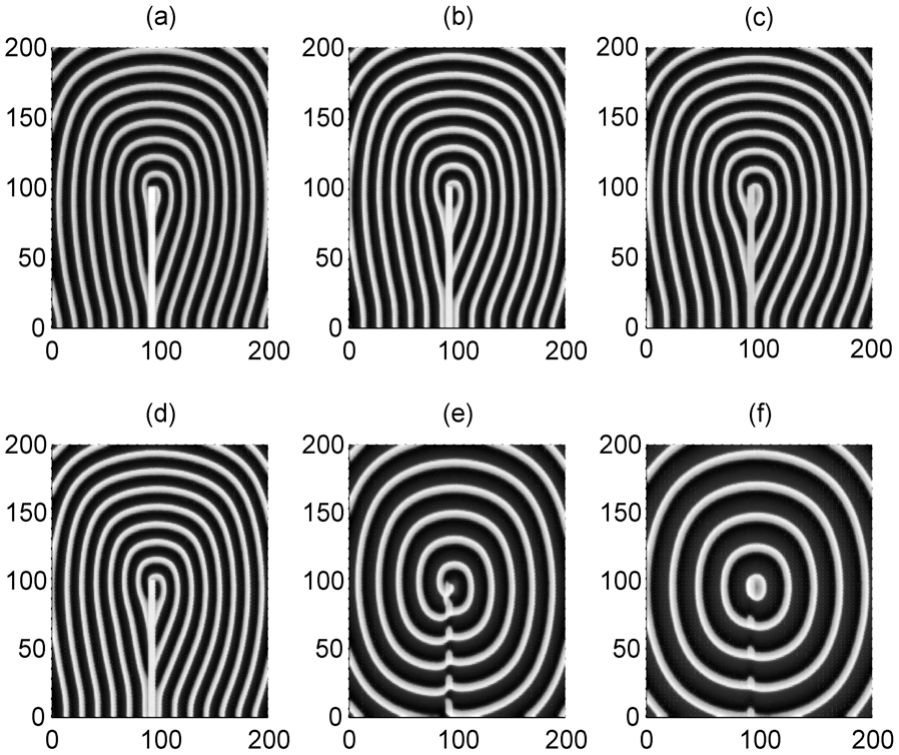
The development of spiral wave under certain channel noise (channel number *N_0_* = 1000) when the target wave is broken by local poisoned area. Blocked area is within 90≤*i* ≤95, 1≤*j* ≤100. The area forced by external currents with diversity is close to the center of the network (90≤*i* ≤95, 90≤*j* ≤95), *I*_0_ = 60,*I*_2_ = 40, for *x*_k_ = 0.1 (panel a) ; *x*_k_ = 0.2 (panel b), *x*_k_ = 0.3 (panel c), *x*_k_ = 0.4 (panel d), *x*_k_ = 0.5 (panel e), *x*_k_ = 0.6 (panel *f*), coupling intensity *D* = 2, transient period *t* = 800 and no-flux boundary condition is used.

The results in Fig. 9 confirm that a spiral wave could also be developed to occupy the network even when channel noise is also present. Then we investigate the development of a pattern when the target wave is broken by a poisoned area in which ion channels are blocked, and the results are shown in Fig. 10.

**Figure 11:**
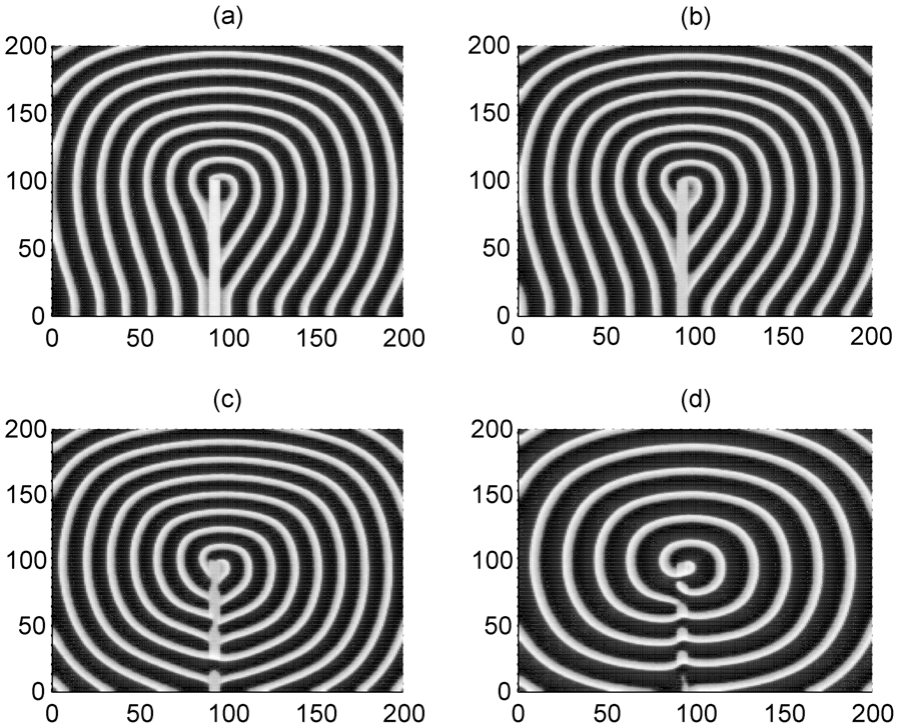
The development of spiral wave under certain channel noise (channel number *N_0_* = 500) when the target wave is broken by local poisoned area. Blocked (poisoned) area is within 90≤*i* ≤95, 1≤*j* ≤100. The area forced by external currents with diversity is close to the center of the network (90≤*i* ≤95, 90≤*j* ≤95), *I*_0_ = 60,*I*_2_ = 40, for *x*_k_ = 0.2 (panel a) ; *x*_k_ = 0.3 (panel b), *x*_k_ = 0.4 (panel c), *x*_k_ = 0.5 (panel d), coupling intensity *D* = 2, transient period *t* = 800 and no-flux boundary condition is used.

Clearly, a distinct spiral wave is also developed under moderate poisioning ratio as shown in Fig.10e, depth poisoning (few ion channels could be open) is helpful to develop the spiral wave when the target wave is blocked due to the effect of channel blocking. Furthermore, we will check another case when the target wave is broken by the local poisoned area in the presence of channel noise, and the results are shown in Fig. 11.

The results in Fig.10 and Fig. 11 confirm that a spiral wave could also be developed when a target wave is broken by the poisoned area due to channel blocking in the presence of channel noise. Extensive numerical results show that breakup of a spiral wave occurs when the channel noise is increased greatly (smaller channel number N being considered).

**Figure 12:**
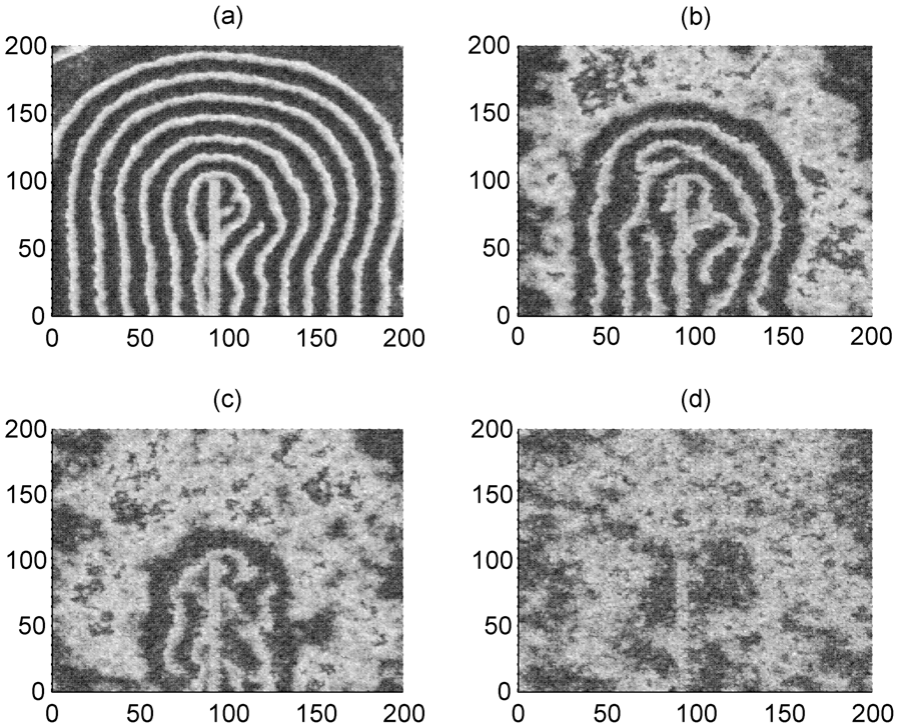
The evolution of spiral wave under different additive Gaussian white noise. Blocked area is within 90≤*i* ≤95, 1≤*j* ≤100, *x*_k_ = 0.3. The area forced by external currents with diversity is within the area 90≤*i*≤95, 90≤*j* ≤95, *I*_0_ = 60, *I*_1_ = 40, for noise intensity *D_noise_ = *100 (panel a) ; *D_noise_ = *200 (panel b) ; *D_noise_ = *300 (panel c) ; *D_noise_ = *500 (panel d), coupling intensity *D* = 2, transient period *t* = 800 and no-flux boundary condition is used.

Furthermore, the effect of additive noise is also detected and discussed, and the results are plotted in Fig. 12. For simplicity, the Gaussian white noise is imposed on the neurons in the network.

The results in Fig. 12 confirm that the developed spiral wave could keep alive under certain additive Gaussian white noise, but breakup of the spiral wave occurs when the intensity of additive noise is increased greatly.

**Figure 13:**
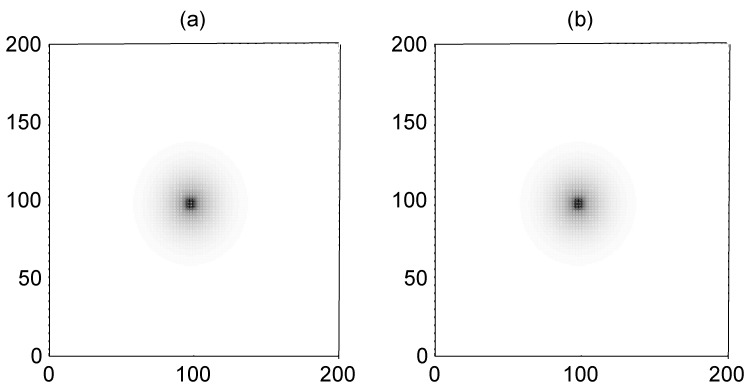
The formed patterns due to blocking in ion channels in calcium. The developed state (*t* = 800 time units) when some channels of calcium in neurons in a square array (95≤*i* ≤100, 95≤*j* ≤100) is blocked at *x*_k_ = 1, coupling intensity *D* = 4, for *x*_Ca_ = 0.1 (panel a), *x*_Ca_ = 0.2 (panel b).

Finally, it is interesting to check the case about pattern formation when ion channels of calcium, the development of target wave due to partial block of calcium ion channels is plotted in Fig. 13.

**Figure 14:**
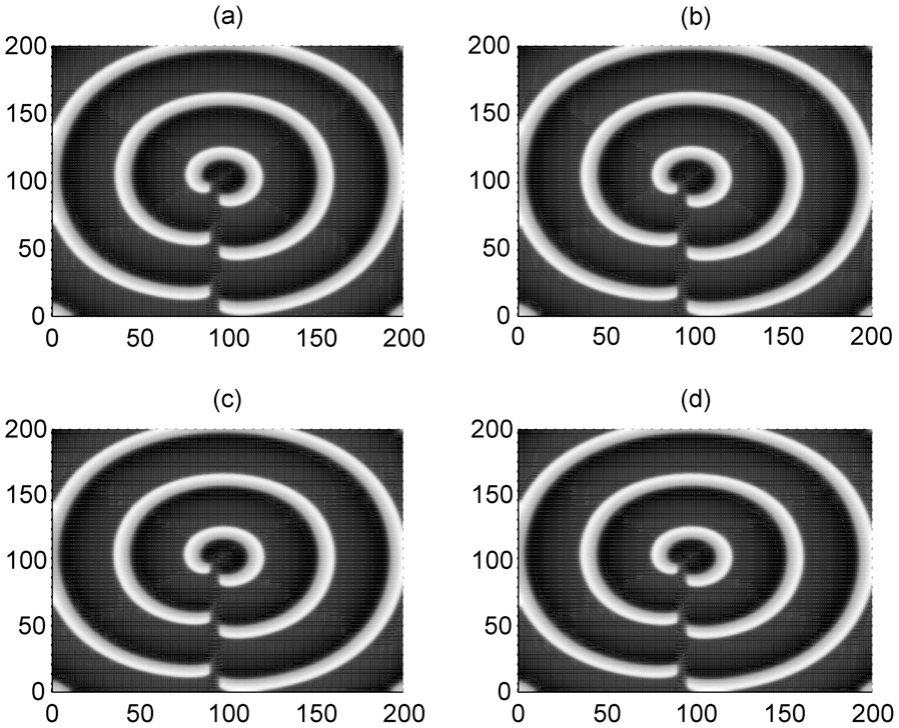
The formed patterns under forcing currents with diversity and blocking in ion channels in calcium. The developed state (*t* = 800 time units) when some channels of calcium in neurons in a square array (90≤*i* ≤95, 1≤*j* ≤100) is blocked at *x*_k_ = 1, for *x*_Ca_ = 0.1 (panel a), *x*_Ca_ = 0.2 (panel b), *x*_Ca_ = 0.3 (panel c) ,*x*_Ca_ = 0.4 (panel d) ; the forcing currents with diversity are imposed on the local area 95≤*i* ≤100, 100≤*j* ≤105 with *I*_0_ = 60, *I*_1_ = 40 for other nodes, coupling intensity *D* = 4.

The results in Fig. 13 confirm that the formed target wave cannot expand but just occupies a local area in the network, even if the calcium ion channels are poisoned greatly (smaller *x*_Ca_). Furthermore, we also investigate the competition between the partial calcium ion channels blocking in a local area and the forcing currents with diversity, and the results are plotted in Fig. 14.

The results in Fig. 14 show that spiral wave could also be induced when the target wave is broken by the poisoned area. It also shows some slight difference from the results for blocking of potassium channels because the fluctuation of membrane potential depends on the calcium conductance and potassium conductance in different orders.

In a summary, we simulated the formation of a target wave by imposing external currents with diversity and partial poisoning of ion channels in a local square area (as shown in Fig. 7). A spiral wave could be developed when the target wave is broken by the artificial defects and/or local poisoned area. The initiation of the spiral wave is robust to certain channel noise and additive Gaussian white noise. In fact, both of the forcing currents with diversity and the poisoning of ion channels in a fraction of neurons in the network can measure some properties of spatial heterogeneity in the network, several types travelling waves will be generated in due to spatial heterogeneity and one kind of travelling waves could suppress others after competition or interaction. More importantly, we confirmed that partial ion channel blocking could generate a similar effect on breaking the target wave to induce spiral wave as the artificial defects. It could indicate that the origin of defects in the network results from channels poisoning in local area.

### Conclusions

In this paper, the development of ordered waves such as target wave and spiral wave in the network of neurons is investigated, and the effect of additive Gaussian white noise and channel noise is also considered, respectively. Our results are summarized as follows.

1. A target wave could be developed to occupy the network by imposing external forcing currents with diversity or blocking partial ion channels of potassium in a fraction of neurons of the network (local poisoned area).
2. A spiral wave could be induced to occupy the network when the target wave is broken by the artificial defects and keeps robust to channel noise and certain additive Gaussian white noise.
3. A spiral wave could be generated in the network when the target wave is blocked by the local poisoned area in which potassium ion channels in a square array are partially poisoned and blocked. The developed spiral wave is also robust to channel noise and additive Gaussian white noise. Breakup of the spiral wave occurs when the intensity of noise is increased greatly.
4. It is confirmed that partial ion channel poisoning or blocking in a fraction of neurons could generate a similar effect of breaking the target wave as the artificial defects; it is argued that the potential origin for defects in the neuronal system could be associated with the partial poisoning in channels of a few neurons in the network. That is to say, the channel blocking could account for the existence of defects in the neuronal system. It could give us new insight into understanding the formation and emergence of a spiral wave in a neuronal system as observed in the cortex of brain.
